# Risk and protective effects of serotonin and BDNF genes on stress-related adult psychiatric symptoms

**DOI:** 10.1016/j.ynstr.2019.100186

**Published:** 2019-07-26

**Authors:** Paul G. Nestor, Keira O'Donovan, Hannah E. Lapp, Victoria Choate Hasler, Sara B. Boodai, Richard Hunter

**Affiliations:** Department of Psychology, University of Massachusetts Boston, Boston, MA, 02125, USA

**Keywords:** Adverse childhood experiences, Risk factors, Early life stress

## Abstract

We focused on individual risk by examining childhood adversity and current psychiatric symptoms in a sample of 100 college students genotyped for both the serotonin transporter (5-HTTLPR) and the brain-derived neurotrophic factor (BDNF). Naturally occurring allelic variation in 5-HTTLPR (short/long) and BDNF (valine/methionine) have been strongly implicated in stress-related psychiatric risk, but the combined effects of these alleles on psychological functioning have yet to be fully elucidated. Univariate analysis revealed gene-environment correlations linking heightened psychiatric risk with past childhood adversity for short but not long 5-HTTLPR allelic carriers and for valine (Val) but not methionine (Met) BDNF allelic carriers. Multivariate analyses revealed a significant gene x gene interaction with results showing that risk varied systematically depending on both 5-HTTLPR and BDNF alleles, independent of childhood adversity. Hierarchical regression analyses indicated that approximately 11% of the variance in symptoms of depression could be specifically accounted for by the epistatic interaction of 5-HTTLPR and BDNF val66Met polymorphisms. Allelic group analyses indicated lowest risk, as measured by depression and anxiety, for allelic carriers of 5-HTTLPR-short and BDNF Met, followed by 5-HTTLPR-long and BDNF-Val, 5-HTTLPR-short and BDNF-Val, and 5-HTTLPR-long and BDNF-Met. Results suggest that protective or risk-enhancing effects on stress-related psychiatric functioning may depend on specific allelic combinations of 5-HTTLPR and BDNF.

## 1 Introduction

The concept of risk originating from influential epidemiological studies of atherosclerotic disease of the 1960s (Dawber, 1980) has long played a central role in models of psychopathology. Traditionally understood in reference to specific and discrete mental illness, risk is now often viewed trans-diagnostically, with the focus on elucidating general mechanisms and underlying etiological and pathophysiological processes and phenotypical precursors that may be common across different psychiatric disorders (Cuthbert and Insel, 2013). From this perspective, whether a particular form of mental illness is ultimately expressed depends on a combination of environmental, genetic, and temperamental factors. Here the emphasis is not on illness per se but on endophenotypes of risk that can be represented at various levels of analyses ranging from molecular genetics to brain structure and function to behavior and psychological traits (Cuthbert and Insel, 2013). These risk endophenotypes are thought to have simpler genetic architectures than more complex and remote clinical phenotypes, including those that characterize the full prodromal states of particular illnesses, such as schizophrenia or bipolar disorder (Canli and Lesch, 2007; Gottesman and Gould, 2003). Endophenotypes of risk may thus be more sensitive to the interacting effects of normal genetic variation and environmental factors on brain structure and function in emerging psychopathology (Gottesman and Gould, 2003). These effects, in turn, may be especially pronounced during late adolescence and early adulthood, a well-known developmental period of risk for mental illness (Hooley et al., 2017).

Depression and anxiety represent key facets of psychiatric risk. For example, behavior genetic studies of identical and fraternal twins have shown moderately high heritability values for emotions such as depression and anxiety (Eley et al., 2004; Stevenson et al., 1992; Stein et al., 1999). Similarly, genetic studies have linked individual differences in these risk-enhancing negative emotions (Parasuraman and Jiang, 2012) to specific allelic variants arising from single nucleotide polymorphisms (SNPs) that are hypothesized to influence neurotrophic or neurotransmitter function (Canli and Lesch, 2007; Parasuraman and Jiang, 2012). These SNPs have in turn been shown to moderate the impact of environmental variables, particularly early life stress, on later emotional functioning in both healthy (Grabe et al., 2012) and clinical (Caspi et al., 2003; Kaufman et al., 2006; Kendler et al., 2005) samples. Together, findings from these studies provide evidence of the endophenotypic plasticity of risk (see Charney, 2012).

Perhaps the most well studied candidate is the gene encoding the serotonin transporter (5-HTT), which contains a regulatory variation linked to stress-related disorders of anxiety and depression (Canli and Lesch, 2007; Lesch et al., 1996; Licinio and Wong, 2001). The 5-HTT protein is encoded by a single gene, *SLC6A4* whose transcriptional activity is modulated by a variable number of tandem repeats in the 5’ promoter region (5-HTTLPR). This repetitive sequence leads to systematic allelic variation of 5-HTT expression and function that has been directly linked to phenotype differences in negative emotions of depression and anxiety. Specifically, the carriers of the low-expressing 5-HTTLPR short allele have elevated risk for depression, particularly in the context of social adversity (Caspi et al., 2003; Eley et al., 2004; Kendler et al., 2005), and also tend to have increased anxiety and stress reactivity, as demonstrated in healthy samples (Canli and Lesch, 2007). In comparison, carriers who are homozygous for the long 5-HTTLPR allele have increased transcriptional activity, and reduced risk for depression, anxiety, and stress reactivity (Canli and Lesch, 2007) as well as heightened levels of optimism (Fox et al., 2009). A more recent meta-analysis, however, failed to find support for increased risk of depression following childhood adversity in short allele carriers of 5-HTTLPR (Culverhouse et al., 2018). In fact, the Culverhouse et al. meta-analysis, which included 31 studies totaling 38,802 individuals of European ancestry, did not replicate the widely-published finding of increased risk of depression following a stressful event for short allele carriers of 5-HTTPLR compared with long allele carriers (Ancelin and Ryan, 2018).

The gene encoding the brain-derived neurotrophic factor (BDNF) has also been closely linked to depression. As a member of the neurotrophin family, BDNF plays a critical role in regulating cellular structure and plasticity in the adult brain (Bramham and Messaoudi, 2005; Lu, 2002; Kovalchuk et al., 2002; Tanaka et al., 2008). Located on human chromosome 11p13, the BDNF gene contains a guanine-to-adenine SNP at nucleotide 196 (rs6265), which results in an amino acid substitution of valine (Val) to methionine (Met) at codon66. This SNP, known as the val66met, changes the 5-prime pro-domain of the human BDNF protein reducing BDNF protein secretion in the brain, and leading to increased vulnerability to depression (Egan et al., 2003; Groves, 2007). In fact, data have suggested that the Met allele of the BDNF gene moderates the relationship of childhood adversity and depressive symptoms in adulthood (e.g., Aguilera et al., 2009). In line with these findings, other studies have linked the Met allele with cognitive difficulties in contrast to protective properties of the Val allele (e.g., Egan et al., 2003). However, similar to 5-HTTLPR research reviewed above, another set of findings examining the role of BDNF in depression has come to a diametrically opposite conclusion regarding risk and protective properties of Val and Met alleles. For example, in a sample of 441 Caucasian Americans, carriers of the Val allele had significantly higher scores on neuroticism, a well-established personality risk factor for depression, than did carriers of the Met allele (Sen et al., 2003). Similarly, another study showed that carriers of the Val/Val allele had higher scores on self-reported anxiety, which often accompanies depression, than did participants with Val/Met or Met/Met genotypes (Lang et al., 2005).

As these foregoing studies show, the roles of 5-HTTLPR and BDNF polymorphisms on mental health risk have been extensively investigated but typically, separately or independent from each other, with relatively few studies examining the joint and interacting effects of these two signaling systems. Indeed, polygenic studies have demonstrated a functional interconnection between serotonin and BDNF pathways in regulating synaptic plasticity and neurogenesis (Martinowich and Lu, 2008). Even more striking are the findings from the relatively few studies that have directly tested for the three-way interaction of 5-HHTTLPR, BDNF, and environmental stress on mental health (e.g., Dalton et al., 2014; Grabe et al., 2012). For example, in a sample of 2035 Caucasian Germans, Grabe et al. (2012) reported that the effect of the short allele of 5-HTLPR on depressive symptoms depended on both BDNF and exposure to environmental stress. Here the results indicated the presence of the 5-HTTLPR short allele and childhood maltreatment did not confer risk when accompanied by the Met allele. In fact, the often demonstrated risk enhancing properties of the short 5-HTLLPR allele in the face of childhood adversity failed to materialize in the presence of the BDNF Met allele. These findings thus underscore multifactorial influences of genes and stress on psychiatric symptoms, offering strong evidence for the role epistatic and environmental interactions may play in mental health risk.

Accordingly, in the current study, we examined both the single and interacting influences of 5-HTTLPR, BDNF, and exposure to adverse childhood experiences (ACE) on individual differences in risk in a healthy sample of college students ranging in ages 18–25 who completed psychiatric symptom ratings, which included separate categories for depression, anxiety, and psychosis. We first examine the genetic contributions of each BDNF and 5-HTTLPR polymorphism, separately, to symptom endophenotypes of psychiatric risk, as measured by psychiatric symptom rating scales. We then employ multivariate analyses to examine interacting influences of the combined effects of these polymorphisms, in conjunction with childhood stress on psychological functioning as assessed by adult psychiatric ratings used as an endophenotype measure of risk.

## 2 Method

### 2.1 Sample and procedures

One hundred participants were recruited from the greater Boston area, primarily at the University of Massachusetts, Boston (UMB). Participants were between the ages of 18 and 25 (M = 21.22 years, SD = 1.99) and identified as English speaking for at least five years prior to study enrollment. Seventy percent of participants identified as biologically female, 42% racially identified as White, 72% reported the United States of America as their country of origin, and 63% endorsed 1–3 years of college as their level of education (see Table 1 for a description of participant characteristics.) The Institutional Review Board (IRB) at UMB approved all research study procedures. Consenting participants completed a self-report demographics questionnaire, the Adverse Childhood Experiences scale (Anda et al., 2006) and the Brief Symptom Inventory (Derogatis and Melisaratos, 1983), Participants were the asked to provide a DNA sample via a cheek swab for the assaying of genotypes. Participants were compensated $25 for their time.

**Table 1:** *Demographic information for full sample (n = 100)*.

	N		Mean *(SD)*
**Biological Sex**		**Age**	21.22 *(1.99)*
Male	30	**Country of Origin**	**N**
Female	70	Belarus	1
**Gender Identity**		Brazil	1
Man	29	China	2
Woman	66	El Salvador	1
Transgender	2	Haiti	3
Other	3	India	9
**Sexual Orientation***(n = 98)*		Iran	1
Bisexual	9	Jamaica	1
Gay/Lesbian	4	Kenya	2
Heterosexual	81	Nepal	2
Other	4	Nigeria	1
**Racial Identity**		Pakistan	1
Asian	20	Saudi Arabia	1
African American/Black	16	Taiwan	1
African American/Indian	1	United States of America	72
Brown	3	Venezuela	1
Caribbean	2	**Highest Level of Education**	
Latin(x)	8	1–3 years of high school	2
Middle Eastern	1	High school diploma	15
Native American	1	1–3 years of college	63
White	42	College degree (BA, BS)	19
White/Latin(x)	4	Graduate degree (MA, MS)	1
White/Middle Eastern	1		
White/Native American	1		

### 2.2 Measures

The Brief Symptom Inventory (BSI) is a 53-item scale that measures psychiatric symptoms status across nine distinct domains: Somatization, Obsessive-Compulsive, Interpersonal Sensitivity, Anxiety, Hostility, Depression, Paranoid Ideation, Psychoticism, and Phobic Anxiety (Derogatis and Spencer, 1982). The BSI also includes measures of overall Global Severity Index (GSI) and a Positive Symptom Distress Index (PSDI). BSI scores reflect current psychiatric status on a Likert-scale ranging from 0 (not at all) to 4 (extremely), where zero indicated the absence of current distress related to psychiatric symptoms. The BSI has demonstrated good internal consistency among non-psychiatric populations (0.71-0.85 across scales) and moderate to high test-retest reliability (0.68-0.91 across scales) and convergent and discriminant validity with the MMPI (Derogatis and Spencer, 1982; Derogatis and Melisaratos, 1983). For the purpose of this study, standardized T scores were used, derived from age-matched normative data, for BSI subscales.

The Adverse Childhood Experiences (ACE) scale (Anda et al., 2006) is a 10-item measure that assesses eight categories of adverse experiences in childhood, including: emotional, physical and sexual abuse, and household dysfunction (i.e., substance abuse, mental illness, mother treated violently, and incarcerated household member). Participants are asked to provide “Yes” or “No” responses to each of the 10 items. Total ACE scores are the sum of affirmative responses to questions such as: “Were your parents ever separated or divorced?” “Did a parent/adult in your household often or very often push, grab, slap, or throw something at you?” “Did a member of your household go to prison?” Each question falls under an initial prompt, specifying the timeframe as the first 18 years of the participants’ life. Scores range from 0 to 10, with higher scores indicative of greater number of adverse events in childhood.

### 2.3 DNA collection and extraction

Cytobrush swabs (Coopersurgical Inc.) were used to collect buccal cells. Participants were instructed to brush the swab 30 times against the inside of their cheek while slowly rotating the swab. Swabs were immediately placed on ice and stored at −80° C until DNA extraction. Buccal samples were extracted using a Zymo Quick DNA Universal Kit per the manufacturer's instructions (Zymo Research). DNA yield from buccal samples ranged from 0.48 μg to 14.4 μg of DNA. Extracted DNA was stored in molecular biology-grade water at −80° C until genotyping analysis.

### 2.4 5-HTTLPR genotyping

Genotyping for 5-HTTLPR polymorphisms was performed using polymerase chain reaction and resolution using gel electrophoresis (adapted from Smith et al., 2004). 25 μL PCR reactions were set up to contain 1X Green GoTaq Flexi Buffer, 1.5 mM MgCl_2_, 0.25 mM PCR Nucleotide Mix, 2.5 ng of DNA sample, and 0.15 μM of both forward and reverse primers (FW: 5′ TGA ATG CCA GCA CCT AAC CC 3′ and RV: 5′TTC TGG TGC CAC CTA GAC GC 3′). DNA amplification was achieved used the following thermocycler programming: Initial denaturation was run for 11 min at 95 °C, followed by 40 cycles of 45 s at 95 °C, 45 s at60 °C, 45 s at 72 °C, and a final elongation step of 72 °C for 10 min. The two amplicon products varied by 44 base pairs (515 base pairs for the long allele and 471 base pairs for the short allele) and were visualized by running the DNA samples on a 1.5% agarose gel stained with 1.5% Ethidium Bromide. Length of amplicon was determined by comparing sample bands to a reference DNA ladder (Promega, USA; ref: G695A) using Molecular Imaging ChemiDoc XRS+. Heterozygous 5-HTTLPR genotype was visibly detected by the presence of two bands in the lane approximately 44 base pairs apart.

### 2.5 BDNF Genotyping

TaqMan SNP genotyping was used to determine BDNF val66met genotype (rs6265). 25 μL PCR reactions were performed using a pre-designed 1X Taqman allelic discrimination assay (Applied Biosystems, USA; assay number: C__11592758_10), containing forward and reverse primers and allele-specific probe with 5 ng of sample DNA. Genotypic amplification was achieved using the StepOne Plus Real-Time (Applied Biosystems) PCR System with programming as follows: 95 °C for 10 min, followed by 42 cycles of 95 °C for 15 s and 60 °C for 1 min. Genotype was determined from the resulting allelic discrimination plot.

For the BDNF gene, there were 72 Val/Val, 21 Met/Met, and 7 Val/Met carriers. We grouped Met/Mets (n = 21) with Val/Mets (n = 7) to form a “Met” genotype (n = 28), with the remaining carriers categorized as “Val/Val” (n = 72) genotype. For the 5-HTTLPR transporter gene, there were 41 Long/Long, 38 Short/Long and 21 Short/Short carriers. The distribution of genotypes followed the Hardy-Weinberg equilibrium for 5-HTTLPR and BDNF alleles (Rodriguez et al., 2009). In addition, we assigned the 41 Long/Long alleles to a 5-HTTLPR transporter-long (“5-HTTLPR-L” n = 41) group, and the remaining 38 Short/Long and 21 Short/Short carriers to a 5-HTTLPR transporter short (“5 HTTLPR-S” n = 59) group. Following previous research (Grabe et al., 2012), we further divided the 100 participants into four allelic groups: 1) 34 5-HTTLPR-L, BDNF Val/Val carriers; 2) 7 HTTLPR–L, BDNF Met carriers; 3) 38 5-HTTLPR-S, BDNF Val/Val carriers; and 4) 21 5HTTLPR-S, BDNF Met carriers.

### 2.6 Statistical analyses

We first submitted ACE scores for each genotype to a 2 × 2 analysis of variance (ANOVA) with two between-subjects factors of BDNF (Val/Val, Met) and 5-HTTLPR serotonin transporter gene (long, short). Next we conducted univariate correlations of ACE and BSI scores for each of the four single gene groups separately. For multivariate group comparisons, BSI scale scores were then submitted to a multiple analysis of variance (MANOVA) with two between-subjects factors of BDNF (Val/Val, Met) and 5-HTTLPR serotonin transporter gene (long, short). This MANOVA tested for the main effects of BDNF and 5-HTTLPR as well as for the interaction of BDNF x 5-HTTLPR on the nine BSI scales. Last as a complement to this MANOVA, a series of hierarchical regression analyses tested for specific and interactional effects of 5-HTTLPR and BDNF on psychiatric risk, namely BSI ratings of depression and anxiety. We entered 5-HTTLPR, BDNF and the interaction of 5HTTLPR x BDNF as predictors of BSI ratings with depression as the dependent variable, and then anxiety. These regression analyses allowed for the quantification of the amount variance in the dependent variable (i.e., depression or anxiety) that could be uniquely accounted for by each of the two independent variables (5-HTTLPR, BDNF) as well as their interaction, 5HTTLPR x BDNF. In all regression analyses, the F-to-enter probability was .05, and F-to exclude probability was .10. Significant levels were two-tailed.

## 3 Results

Participants reported on average 2.17 (SD = 2*.*31) exposures to adverse childhood experiences, with a median of 1.00. The ACE results indicated that 51% (n = 51) of the full sample had no more than one adverse childhood experience, and 24% (n = 24) of the sample experienced four or more adverse events in childhood. For the BSI, highest T-scores occurred for Psychoticism (M = 61.72, SD = 11.96), Obsessive-Compulsive (M = 61.49, SD = 12.66), and Depression (M = 60.45, SD = 11.12), with lowest scores for Somatization (M = 55.32, SD = 11.83) and Anxiety (M = 56.43, SD = 13.16). Table 2 presents ACE and BSI scores for the genotype groups.

**Table 2:** ACE and BSI scores for genotypes: somatization (SOM), obsessive-compulsive (O–C), interpersonal stress (IS), depression (DEP), anxiety (ANX), hostility (HOS), phobia (PHOB), paranoia (PAR), and PSY (psychoticism).

		Single Gene				Allelic Group			
		**5-HTTLPR Long***(n = 41)*	**5-HTTLPR Short***(n = 59)*	**BDNF Val/Val***(n = 72)*	**BDNF Met***(n = 28)*	**5-HTTLPR-L|BDNF Val/Val***(n = 34)*	**5-HTTLPR-L| BDNF Met***(n = 7)*	**5-HTTLPR-S| BDNF Val/Val***(n = 38)*	**5-HTTLPR-S|BDNF Met***(n = 21)*
**ACE**		2.63 ± 2.40	1.85 ± 2.20	2.24 ± 2.29	1.93 ± 2.37	2.41 ± 2.32	3.71 ± 2.62	2.13 ± 2.28	1.33 ± 2.01
**BSI**	SOM	54.90 ± 10.43	55.81 ± 12.79	56.04 ± 11.88	53.46 ± 11.70	54.03 ± 10.07	59.14 ± 11.94	57.84 ± 13.17	51.57 ± 11.27
	O–C	61.71 ± 12.37	61.34 ± 12.95	61.82 ± 12.49	60.64 ± 13.28	59.56 ± 12.31	72.14 ± 5.79	63.84 ± 12.45	63.84 ± 12.45
	IS	61.27 ± 10.94	58.76 ± 13.01	61.49 ± 12.33	55.43 ± 10.93	60.74 ± 11.51	63.86 ± 7.80	62.16 ± 13.15	52.62 ± 10.49
	DEP	61.34 ± 9.51	59.83 ± 12.15	61.89 ± 10.95	56.75 ± 10.87	60.03 ± 9.52	67.71 ± 6.90	63.55 ± 11.98	53.10 ± 9.43
	ANX	57.61 ± 11.71	55.61 ± 14.11	58.18 ± 13.23	51.93 ± 12.64	56.91 ± 11.95	61.00 ± 10.65	59.32 ± 14.35	48.90 ± 11.10
	HOS	58.22 ± 10.28	56.27 ± 11.43	58.36 ± 10.83	53.75 ± 10.78	57.12 ± 10.75	63.57 ± 5.32	59.47 ± 10.92	50.48 ± 10.17
	PHOB	59.00 ± 10.62	56.29 ± 10.98	57.82 ± 11.40	56.32 ± 9.46	58.82 ± 10.78	59.86 ± 10.57	56.92 ± 11.99	55.14 ± 9.02
	PAR	59.83 ± 11.10	57.29 ± 12.24	59.63 ± 11.80	55.00 ± 11.29	59.26 ± 11.23	62.57 ± 10.78	59.95 ± 12.43	52.48 ± 10.51
	PSY	62.49 ± 11.46	61.19 ± 12.37	62.51 ± 12.36	59.68 ± 10.81	61.32 ± 11.98	68.14 ± 6.47	63.58 ± 12.76	56.86 ± 10.57

### 3.1 Single gene

We first examined ACE scores for each genotype. A 2 × 2 analysis of variance (ANOVA) of ACE scores with two between-subjects factors of BDNF (Val/Val, Met) and 5-HTTLPR serotonin transporter gene (L,S) revealed a significant main effect for 5-HTTLPR, F (1, 96) = 5.61, p = .02, Partial Eta Squared = 0.055, with the BDNF x 5-HTTLPR interaction approaching significance, F (1, 96) = 3.49, p = .065, Partial Eta Squared = 0.035. The main effect for 5-HTTLPR reflected higher reported exposure to adverse childhood experiences, as measured by ACE scores for 5-HTTLPR-L (M = 2.63, SD = 2.40) carriers in comparison to 5-HTTLPR-S (M = 1.85, SD = 2.20) carriers. For the BDNF gene, ACE scores did not differ significantly for Val/Val (M = 2.26, SD = 2.29) and Met (M = 1.93, SD = 2.37) carriers.

Table 3 presents ACE correlations with BSI scores for each genotype. As seen in Table 3, for Val/Val carriers, increased exposure to adverse childhood experiences correlated significantly with higher symptoms scores for obsessive compulsiveness, hostility, phobic anxiety, paranoid ideation, global severity, positive symptoms, and positive symptom distress. By contrast, only the BSI domain of hostility correlated with ACE scores for Met carriers. For 5-HTTLPR serotonin transporter gene, increased exposure to childhood adverse experiences for 5-HTTLPR-S carriers correlated significantly with all but two BSI domains, specifically with somatization, obsessive compulsiveness, depression, anxiety, hostility, phobic anxiety, psychoticism, global severity, positive symptoms, and positive symptom distress. By contrast, for 5-HTTLPR-L carriers, only obsessive compulsiveness correlated significantly with ACE scores (see Table 3).

**Table 3:** Correlations of ACE and BSI for BDNF and 5-HTTLPR genotypes.

	BDNF Val/Val *(n = 72)*	BDNF Met *(n = 28)*	5-HTTLPR Long *(n = 41)*	5-HTTLPR Short *(n = 59)*
**Brief Symptom Inventory**				
Somatization	.181	.131	.032	***.274***^a^
Obsessive-Compulsive	***.377***^b^	.237	***.308***^a^	***.363***^b^
Interpersonal Sensitivity	.186	.080	.056	.219
Depression	.217	.339	.121	***.334***^b^
Anxiety	.219	.334	.192	***.287***^a^
Hostility	***.232***^a^	***.393***^a^	.118	***.382***^b^
Phobic Anxiety	***.277***^a^	.179	054	***.374***^b^
Paranoid Ideation	***.251***^a^	-.154	-.015	.236
Psychoticism	.160	.181	.024	***.263***^a^
Global Severity Index (GSI)	***.296***^a^	.270	.213	***.330***^a^
Positive Symptom Total (PST)	***.301***^b^	.191	.162	***.320***^a^
Positive Symptom Distress Index (PSDI)	***.341***^b^	.283	.248	***.369***^b^

a Correlation is significant at the 0.05 level (2-tailed).

b Correlation is significant at the 0.01 level (2-tailed).

### 3.2 Allelic groups

**Fig. 1:**
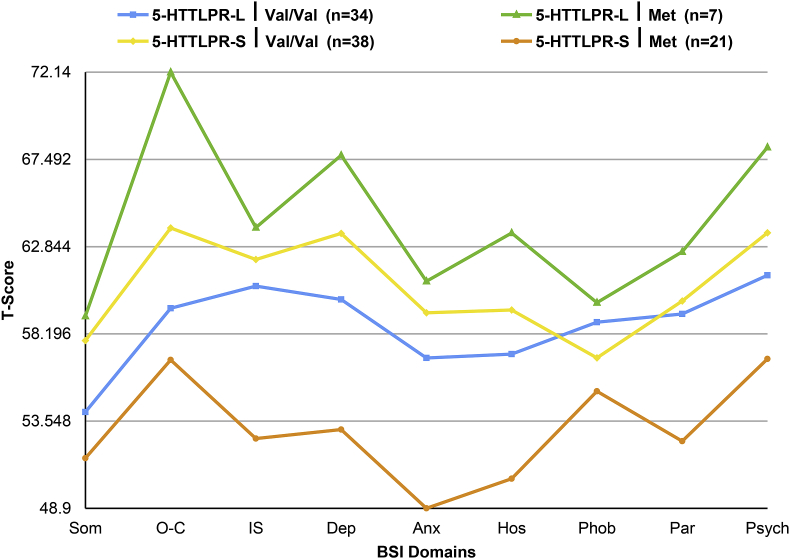
BSI ratings of somatization (SOM), obsessive-compulsive (O–C), interpersonal stress (IS), depression (DEP), anxiety (ANX), hostility (HOS), phobia (PHOB), paranoia (PAR), and PSY (psychoticism) for the four allelic groups.

Fig. 1 plots BSI ratings for the four allelic groups. As shown in Fig. 1, the 5-HTTLPR-S, Met carriers had the lowest level of symptoms, followed by the 5-HTTLPR-L, Val/Val carriers, then by the 5-HTTLPR-S, Val/Val carriers, with 5-HTTLPR-L, Met carriers endorsing highest psychiatric symptoms. Submitting these BSI ratings to a multivariate analysis of variance (MANOVA) with two between-subjects factors of 5-HTTLPR (long, short) and BDNF (Val/Val, Met) revealed a significant 5HTTLPR x BDNF interaction, F (9, 88) = 2.35, p = .02, Partial Eta Squared = 0.194, which remained significant when covarying for adverse childhood experiences, F (9, 87) = 1.98, p = .05, Partial Eta Squared = 0.170. A follow-up MANOVA with only BSI measures of anxiety and depression revealed a highly statistically significant 5HTTLPR x BDNF interaction, F (2, 94) = 4.94, p = .02, Partial Eta Squared = 0.095. Planned comparisons indicated lowest stress-related risk (BSI ratings of depression, anxiety) for 5-HTTLPR-S Met (n = 21) carriers relative to 5-HTTLPR-S, Val/Val (n = 38) carriers, F(2,56) = 5.88, p = .005, Partial Eta Squared = 0.173, 5-HTTLPR-L, Val/Val (n = 34) carriers, F(2,52) = 3.97, p = .025, Partial Eta Squared = 0.173, and 5-HTTLPR-L, Met (n = 7) carriers, F(2,25) = 6.79, p = .004, Partial Eta Squared = 0.352 (see Table 2).

Last, a series of hierarchical regression analyses tested for main and interactional effects of 5-HTTLPR and BDNF on psychiatric risk. 5-HTTLPR, BDNF and the interaction of 5HTTLPR x BDNF were each entered as predictors of BSI ratings of depression. Only the interaction of 5HTTLPR and BDNF accounted (Beta = −0.688, t = −3.52, p = .001) for a significant portion of the variance in depression. For this interaction, partial correlation value of −0.338 and semi-partial correlation value of −0.331 indicated that approximately 10.96%–11.42% of the variance in depression could be explained by serotonin-BDNF interaction. Likewise, only the interaction of 5HTTLPR and BDNF contributed significantly to anxiety (Beta = −4.51, t = −2.30, p = .024), accounting for approximately 4.97%–5.24% of the variance in rating scores for this symptom. Thus, these hierarchical regression analyses were consistent with results derived from MANOVA.

## 4 Discussion

We examined individual differences in psychiatric risk in a sample of 100 college students who were genotyped for 5-HTTLPR and BDNF polymorphisms and who completed measures of childhood adverse experiences and current psychiatric symptomatology. We examined single, joint and interacting influences of these two a priori selected genes on stress-related psychiatric risk. Early studies have traditionally focused on how a single genotype may moderate environmental factors in predicting a behavioral outcome. Here we extended this focus to examine joint and epistatic (gene-gene interaction) influences of two genotypes, BDNF and 5-HTTLPR on the relationship of environmental stress and psychiatric symptoms.

First with regard to the serotonin transporter gene, univariate analyses demonstrated that for participants with either one or two copies of the 5-HTTLPR short variant, increased exposure to adverse childhood events correlated significantly with higher levels of psychiatric symptomatology across several domains, including depression, anxiety, and psychoticism. By comparison, for participants who were homozygous for the HTTLPR long variant, the data revealed no evidence of gene-environment correlation with the sole exception of childhood adversity and obsessive compulsiveness. This finding occurred despite higher overall adverse childhood experiences for the long variant relative to the short variant. Such a pattern of gene-environment correlations is consistent with population and family-based genetic studies, linking the long variant as protective against early exposure to social stress, and the low-expressing 5-HTTLPR short variant as increasing risk for affective spectrum disorders (e.g., see Canli and Lesch, 2007; Uher and McGufin, 2008).

Correlational data also showed that the BDNF genotype influenced the impact of early life stress on later mental health functioning. However, the pattern of these BDNF-environment correlations did not conform to what would be expected on the basis of many prior studies. That is, several lines of evidence have suggested that the substitution of methionine for valine renders methionine carriers of the BDNF gene more vulnerable to anxiety, depression, and general psychiatric risk within the context of early life stress (Aguilera et al., 2009; Hayden et al., 2010; Kaufman et al., 2006), although others have failed to find such relationship (e.g., Willis-Owen et al., 2005). The same literature suggests that valine, by comparison, confers an advantage, reducing risk of experiencing negative emotionality in the face of social adversity for valine homozygote carriers (e.g., Bath and Lee, 2006; Duman, 2004). Yet, in the current study, strongest gene-environment correlational effects occurred for valine homozygotes relative to methionine carriers, suggesting that the valine carriers were more psychologically vulnerable to childhood stressful events than those who had a least one copy of the Met allele of the BDNF gene. These data suggested that the Met allele but not the hypothesized Val allele may be protective in depression and anxiety --- and this finding is consistent with those of other studies showing the Met allele to be protective for bipolar disorder (Neves-Pereria et al., 2002; Sklar et al., 2002).

Second, the results suggested that individual differences in risk as measured by symptoms of depression and anxiety may be due to the interaction of specific 5-HTTLPR and BDNF alleles. This statistically significant gene x gene interaction of 5-HTTLPR and BDNF occurred, independent of childhood stress, and made a specific and unique contribution to the expression of risk, accounting for approximately 11% of the variability in depression symptoms, as demonstrated by hierarchical regression analyses. This epistasis interaction suggested that risk, as measured by psychiatric symptoms, particularly depression and anxiety, varied systematically depending on both 5-HTTLPR and BDNF alleles. That is, the current results showed that the BDNF Met allele had a risk-reducing effect when paired with the low-expressing 5-HTTLPR-S variant, but a risk-enhancing effect when coupled with the 5-HTTLPR-L variant. On the other hand, the BDNF valine had a consistent risk-enhancing effect when accompanied by either the short or the long variant of 5-HTTLPR. Thus, the most striking and novel findings of the current study pointed to a protective role of BDNF-Met, with this allele mitigating, if not reversing the risk effects of the 5-HTTLPR-S, which have been so well-documented in single-gene studies. However, recent meta-analytic evidence has called into question the reliability and replicability of findings of single gene studies showing increased risk for depression in the face of childhood adversity for short allele carriers of 5-HTTLPR compared with long allele carriers of 5-HTTLPR (see Culverhouse et al., 2018). Our current findings suggested that interacting effects of 5-HTTLPR and BDNF may be a critical factor in understanding the genetic architecture of psychiatric risk. Future studies examining joint and interacting influences of these two genes may help form the necessary meta-analytic evidence for testing the 5-HTTLPR x BDNF polymorphism interaction hypothesis of stress-related psychiatric risk.

5-HTTLPR and BDNF alleles have typically been studied separately, with investigations testing how polymorphisms in either of these two genes may moderate the relationship of early life stress and adult psychiatric risk. Relatively fewer studies (see Uher and McGufin, 2008), however, have investigated the interacting influences of 5-HHTLPR and BDNF in combination with early life stress on adult psychiatric risk (e.g., Aguilera et al., 2009; Kaufman et al., 2006; Nederof et al., 2010; Wichers et al., 2008). These studies include those that have examined the combined effects of 5-HTTLPR and BDNF alleles on behavioral traits (Jiménez-Treviño et al., 2019), brain structure and depression (Han et al., 2018), functional brain activity and emotional reactivity (Wang et al., 2012) and childhood adversity (Benedetti et al., 2017). Most relevant to the current investigation is a population-based study by Grabe et al. (2012) that showed that a three-way interaction of 5-HTTLPR, BDNF and childhood adversity moderated susceptibility to adult depression. In this gene-by-gene-by-environment interaction, Grabe et al. found reduced risk of depression for carriers of BDNF Met and 5-HTTLPR-S alleles who reported a history of childhood maltreatment. By contrast, the same data pointed to heightened risk of depression for carriers of BDNF Met and 5-HTTLPR-L alleles with histories of childhood abuse. In line with Grabe et al., our data showed a significant gene-by-gene interaction with BDNF Met reducing risk when paired with the 5-HTTLPR-S variant but increasing risk when paired with the 5-HTTLPR-L variant. However, these effects were independent of childhood adversity, as our data failed to demonstrate the three-way interaction among BDNF, 5-HTTLPR and early life stress. This may reflect a Type 2 error related to limited statistical power of our study sample. It may also reflect that the ACE scores had a negatively skewed distribution, as the sample had relatively few early life stress experiences. Perhaps not surprisingly given these very low ACE scores, the results failed to demonstrate gene x environment interaction.

The current study relied on a sample of non-help seeking college students who fell in the age range of late adolescence to early adulthood. That the average age of onset of many adult psychological disorders occurs during this developmental period (e.g., Blakemore, 2018; Hooley et al., 2017), may have accounted for the adequate distribution of BSI scores with most falling in expected±one standard deviation of the normative sample (M = 50, SD = 10). On the other hand, however, age, which has emerged as an important factor influencing genetic expression of stress reactivity, has often been overlooked in study designs that have generally been limited to adolescents or young adult samples (Ancelin and Ryan, 2018). In a notable exception, Ancelin et al. (2017) recently examined the effects of extrinsic stress and intrinsic stress (diurnal cortisol secretion) on current depression in a longitudinal, population-based study of 334 participants, age 65 or older, genotyped for 5-HTTLPR. Ancelin et al. reported that the effects of extrinsic and intrinsic stress on depression varied according to 5-HTTLPR genotype in their sample of community dwelling elderly. That is, contrary to typical studies of younger populations pointing to the short allele of 5-HTTLPR as a risk factor for depression, Ancelin et al. reported that their results showed HTTLPR-long carriers to be highly vulnerable to extrinsic stress, as assessed by self-report measures of recent stressful events, and were more likely to be diagnosed with recurrent depression. By contrast, for 5-HTTLPR-S carriers, only the intrinsic stress indicator of morning cortisol was associated with depression (Ancelin et al., 2017). Thus, these findings along with other research studies (e.g., Mueller et al., 2011; Uher and McGufin, 2008) underscore the need for sampling a wide range of ages that will allow for critical sub-group comparisons to test for different developmental risk trajectories of gene-by-gene-by-environment interactions in the etiology of mental illness.

Taken together, the current findings provided support for the role of specific interactions of BDNF and serotonin alleles in stress-related psychiatric risk. The findings further suggested that these epistatic interactions may represent an important source of a genetic diathesis or vulnerability for negative emotions, particularly anxiety and depression that in turn constitute well-known stress-related psychiatric risk factors. However, the current findings were not entirely consistent with the diathesis-stress perspective, particularly for our genetic epistatic effects that demonstrated that the 5HTTLPR short variant, regarded as a vulnerability allele due to its well established empirical link to adult depression, actually proved to be protective against psychiatric risk when accompanied by BDNF Met allele. Yet, more recent research has proposed the *differential susceptibility to environmental influences* perspective, as an alternative to the diathesis-stress model. From this perspective, commonly regarded vulnerability genes, such as the 5-HTTLPR short variant, are recast as “plasticity” genes that are hypothesized to increase responsiveness to both positive and negative environmental conditions (Belsky et al., 2007; Belsky and Beaver, 2011; Belsky and Pluess, 2009; Belsky et al., 2013). That is, particular individuals vary in their *plasticity* or susceptibility to environmental influences and these influences can be either good or bad (e.g., Fox et al., 2011). Empirical support for this perspective comes from data linking differential environmental susceptibility to a set of plasticity genes including 5-HTTLPR, a dopamine receptor gene, DRD4, as well as genes responsible for encoding BDNF (Belsky et al., 2007; Brody et al., 2016; Masarik et al., 2014; Belsky and Pluess, 2009).

However, in the current study, we examined only two genes and therefore did not calculate a polygenic plasticity index which typically requires summing three or more alleles. Likewise, we examined only negative environmental experiences, but a direct test of the differential susceptibility hypothesis requires measures of both negative and positive environmental influences. Nevertheless, our principal findings are consistent with the differential susceptibility hypothesis to the following extent. That is, our results showed that carriers of two putative risk alleles (5-HTTLPR short/BDNF Met) had the lowest level of risk and also had the lowest exposure to adverse childhood experiences, although whether they also had increased exposure to positive events is of course unknown. Future studies will need to test this relationship using specific measures of positive environmental influences as well as with genetic assays that include more than two putative plasticity alleles employed in the current investigation. Such studies may contribute to the development of individualized risk profiles that reflect normal genetic variation in particular neurotrophic and neurotransmitter interactions, moderated by both positive and negative childhood experiences, and linked to adult emotional functioning.
